# Chemical and physical equilibria shape dual ice-nucleation pathways in an organic crystal

**DOI:** 10.1038/s42004-026-02086-4

**Published:** 2026-06-06

**Authors:** Galit Renzer, Dawson Bell, Ingrid de Almeida Ribeiro, Kaden Shaw, Mischa Bonn, Valeria Molinero, Konrad Meister

**Affiliations:** 1 Max Planck Institute for Polymer Research, Mainz, Germany; 2 Department of Chemistry and Biochemistry, Boise State University, Boise, ID USA; 3 Department of Chemistry, The University of Utah, Salt Lake City, UT USA

**Keywords:** Environmental chemistry, Chemical physics

## Abstract

Organic crystals critically influence ice formation in natural environments, yet the molecular mechanisms of their ice nucleation activity remain poorly understood. Here we reveal that the exceptional freezing efficiency of phloroglucinol (PGL), a simple polyhydroxylated aromatic compound, arises from a dynamic interplay of chemical and physical equilibria that generate two concurrent nucleation pathways. One pathway originates from crystalline PGL surfaces that act as potent ice templates, whereas the second occurs in solution, where dissolved molecules assemble into nanoscale aggregates capable of nucleating ice even below the solubility limit. We find that alkaline pH eliminates both pathways by inducing tautomeric shifts that alter hydrogen bonding motifs and suppress molecular assembly. Aging in solution diminishes ice-nucleating activity, likely through oxidative or polymerization processes, while freeze-thaw cycling partially restores activity by generating fresh PGL crystals. These results identify molecular structure, solubility, pH-dependent tautomerism, and phase behavior as key determinants which collectively control ice nucleation, offering a generalizable framework for understanding and predicting the activity of phenolic and other organic ice nucleators in complex environmental settings encountered in atmospheric and cryobiological systems.

## Abstract (web summary)

Organic crystals influence ice formation in natural environments, but the underlying mechanisms remain underexplored. Here, the authors show that the freezing efficiency of phloroglucinol (PGL) stems from nucleation pathways involving its crystalline surface — which acts as an ice template — and dissolved molecules in solution that assemble into nanoscale aggregates capable of nucleating ice even below the solubility limit.

## Introduction

The freezing of water is one of the most fundamental and widespread phase transitions on Earth. It plays a central role in atmospheric processes, cryobiology, and environmental chemistry^1–5^, and proceeds primarily through heterogeneous nucleation by particles that function as ice nucleators (INs)^6^. Organic compounds represent a chemically diverse and highly efficient class of INs that are abundant in aerosols^7–9^, soils^10–12^, and biological fluids^13,14^, where they occur in both crystalline and dissolved states. This group includes amino acids^15,16^, carboxylic acids^17,18^, polyphenols^19,20^, and polyhydroxylated aromatic compounds^17,18,21–24^, many of which originate naturally from metabolic processes^25–27^ or result from the degradation of plant biomass^27,28^ and combustion products^29,30^. These molecules contribute not only as individual INs but serve also as structural building blocks of larger ice nucleation-active macromolecular systems such as humic-like substances^31^, lignin-derived aerosols^7^, and secondary organic matter^32^.

Despite their ubiquity, the molecular origin of ice nucleation by organic compounds remains poorly understood. Unlike inorganic materials, where lattice matching and surface polarity are well-established determinants of nucleation efficiency^33–35^, the chemical motifs and physicochemical features governing ice nucleation by organic INs remain unclear. Organic compounds possess flexible structures, accessible protonation equilibria, and multiple phase states that challenge traditional structure-activity concepts and raise fundamental questions about how molecular interactions, phase transitions, and environmental variables control their ice nucleation activities.

**Fig. 1:**
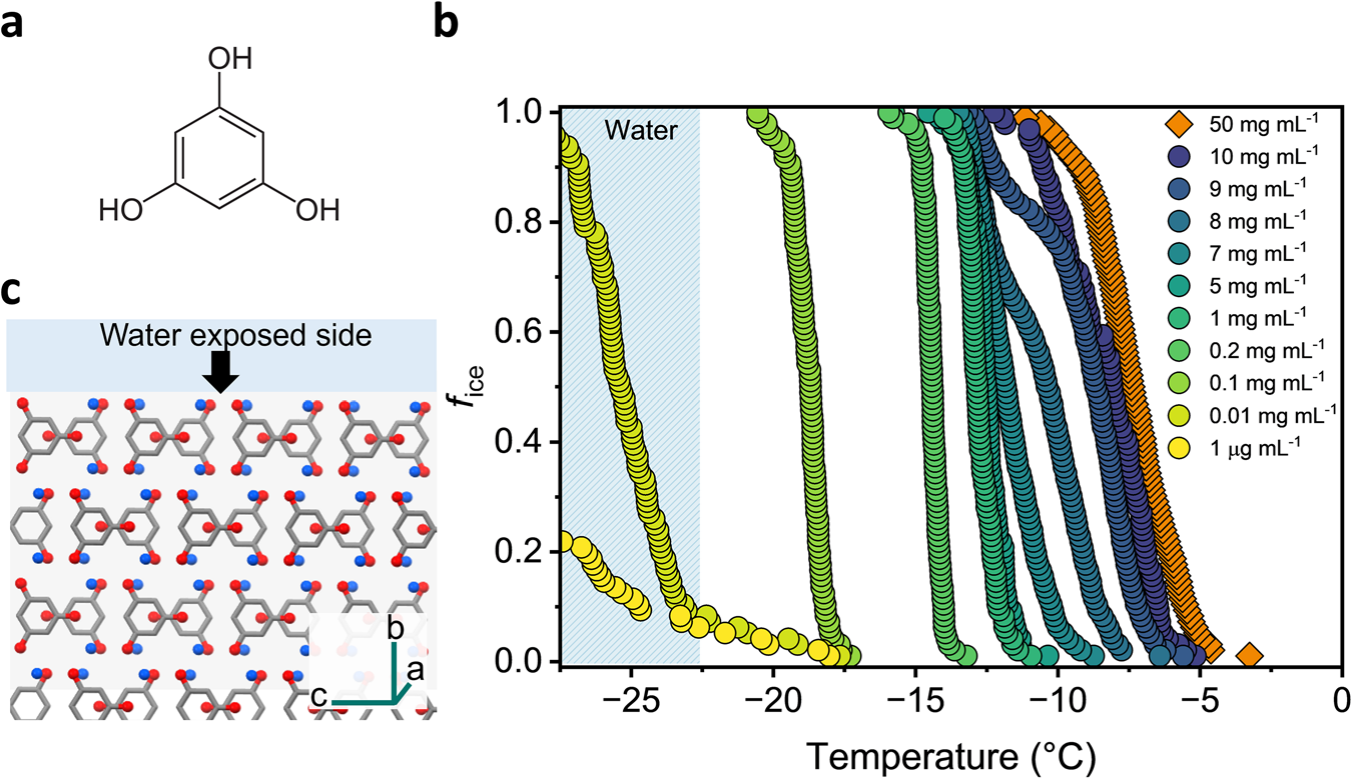
Ice nucleation measurements of aqueous PGL solutions. **a** Molecular structure of Phloroglucinol. **b** Fraction of frozen droplets *f*_ice_ for PGL solutions ranging from 50 mg mL^−^^1^ to 1 µg mL^−1^. The blue-shaded region represents the freezing temperature of water in our system (< −23°C). **c** Schemati**c** of the water-exposed (010) crystal face of a PGL dihydrate crystal. Red and blue balls represent the hydroxyl groups of PGL and water molecules in the crystal, respectively.

Phloroglucinol (PGL, 1,3,5-trihydroxybenzene) is a prime example (Fig. 1a). Due to its structural simplicity paired with high ice nucleation efficiency, PGL has attracted sustained attention for more than sixty years, making it an ideal model system to address these questions. In the early 1960s, Langer and co-workers reported that crystalline PGL could trigger freezing at temperatures as high as −2 °C^22^, yet subsequent studies reported widely varying freezing temperatures depending on particle size, hydration state, and dispersion, and confirmed that PGL crystals can act in both immersion and contact freezing modes^22,24,36–40^. Evans proposed that nucleation might proceed through an ordered monolayer of interfacial water on PGL dihydrate crystal surfaces, representing a deviation from the classical nucleation pathway^40,41^. Molecular simulations by Molinero and colleagues demonstrated that while PGL crystals can promote ordering of interfacial water, the rate-limiting step is still the formation of the initial ice crystallite^42^, consistent with a classical nucleation mechanism in which the structured water layer facilitates rather than determines the nucleation process. However, none of these models explain the pronounced variability in nucleation activity or the sensitivity of PGL to environmental conditions such as pH, concentration or chemical alterations.

These inconsistencies point to a more fundamental mechanistic gap because organic INs do not exist as static entities. Their chemical speciation (e.g., tautomerism and ionization states), solubility, aggregation, and phase behavior vary on experimentally relevant timescales and can generate multiple, coexisting nucleating species. The role of such coupled chemical and physical equilibria in shaping ice nucleation has remained largely unexplored, and the lack of mechanistic insight limits our ability to predict or model the freezing behavior of organic INs.

Here we show that PGL’s ice nucleation activity is controlled by a complex interplay of dynamically shifting equilibria in which molecular structure, tautomeric state, solubility, and phase behavior act together to determine its freezing efficiency. Using high-throughput droplet freezing assays, Raman spectroscopy, dynamic light scattering (DLS), and classical nucleation theory, we study both crystalline and dissolved states and identify two distinct nucleation pathways that coexist, involving crystalline surfaces and nanoscale solution aggregates. Because these molecular characteristics are shared widely among phenolic and polyhydroxylated compounds, our findings provide a general framework for understanding and predicting the ice nucleation activity of organic materials across atmospheric and cryobiological environments, where chemical and physical equilibria fluctuate continuously.

## Results

### Concentration-dependent freezing reveals more than one ice-nucleating species in PGL solutions

Droplet freezing experiments of PGL solutions reveal freezing behavior that cannot be attributed to a single, static ice-nucleating entity. In general, PGL solutions exhibit concentration-dependent freezing, with freezing temperatures shifting to colder values upon dilution (Fig. 1b). Notably, at concentrations ≥5 mg mL^−1^, PGL solutions exhibit two distinct modes of activity, with freezing events clustering around ~−7 °C and ~−12 °C. The relative prominence of these modes shifts systematically with concentration. At high concentrations, the warmer temperature mode dominates, showing freezing efficiencies close to that of the highly potent crystalline phase. Dilution weakens this mode in intensity until it eventually disappears, leaving only the colder mode active. The crystalline phase was tested as an oversaturated solution of 50 mg mL^−1^, where undissolved crystals are expected to coexist with the dissolved fraction. The particle size distribution (Suppl. Fig. S1) shows that the crystals exhibit a mean diameter on the order of approximately 100 µm, confirming the presence of micrometer-scale crystals. Previous molecular simulations identified the (010) face as the one responsible for ice nucleation by large PGL dihydrate crystals^42^. That face is the most efficient ice-binding site (Fig. 1c), exhibiting the smallest lattice mismatch to ice and producing nucleation temperatures consistent with experiments^7,42^. Although direct crystallographic characterization was constrained by technical limitations (see Methods), the observed nucleation activity of crystals, in strong agreement with simulation results, clearly supports that micrometer-scale PGL dihydrate crystals exposing the (010) face are the source of the warm nucleation mode.

This bimodal behavior in solution indicates the presence of at least two subpopulations of different efficient ice-active species and resembles that of bacterial INs, where discrete temperature modes correspond to structurally distinct classes of protein aggregates differing in size and assembly state^43^. To investigate the underlying IN classes in PGL solutions, we calculated the cumulative number of ice nucleation sites per unit mass (*N*_m_) using the Vali formula^44^ (Suppl. Fig. S2). We find that *N*_m_(*T*) spectra of PGL solutions did not overlap across concentrations, in contrast to the expected behavior for systems with a stable IN population, displaying a continuous freezing profile. In such systems, serial dilution typically enables detection of nucleators with different freezing efficiencies by separating them into individual droplets without altering their properties. Instead, each PGL concentration produced a unique freezing profile, indicating that dilution alters the population of ice-nucleation-active structures. These results suggest that PGL does not generate a fixed population of nucleators but forms a dynamic concentration-dependent ensemble of species in solution, whose ice nucleation activity might be modulated by aggregation, chemical equilibria, solubility limits, or phase transitions.

### Tautomeric and protonation equilibria govern ice nucleation activity

**Fig. 2:**
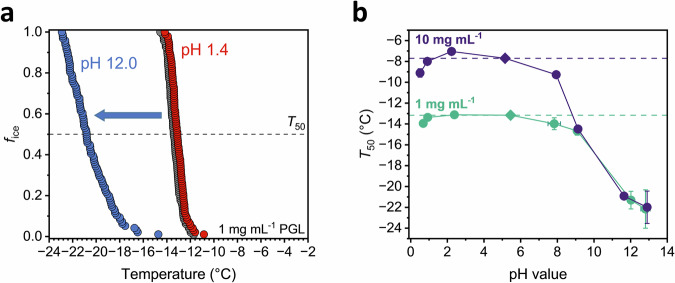
Ice nucleation measurements of aqueous PGL solutions across different pH values. **a** Fraction of frozen droplets *f*_ice_ of 1 mg mL^−1^ PGL solution (grey, behind the red data) at highly basic (blue) and acidic (red) conditions. The dotted line represents the *T*_50_ values, at which 50% of droplets are frozen. **b** *T*_50_ values (circles) of 10 mg mL^−1^ and 1 mg mL^−1^ PGL solutions as a function of pH level. Diamonds represent the IN activity at the standard pH. Data from three pH measurements are presented as the mean ± standard deviation.

The strong concentration sensitivity of PGL’s nucleation activity and the presence of various subpopulations suggest that multiple molecular states may contribute. pH-dependent freezing experiments confirm that PGL’s ice nucleation activity critically depends on its tautomeric form and protonation state. As shown in Fig. 2a, the freezing behavior of PGL at acidic (pH 1.4) resembles that at standard conditions (pH 5.5), while it markedly decreases at alkaline conditions (pH 12.0). Systematic pH probing from 2 to 14 provides a more detailed understanding of this effect and reveals that this decline in freezing efficiency, expressed as *T*_50_ values, the temperature at which 50% of droplets are frozen, occur as a stepwise process. At pH ≥ 12, the PGL solutions no longer exhibit ice nucleation activity. In contrast, acidic conditions have negligible effects on the freezing behavior. Further, both PGL subpopulations, tested at 1 and 10 mg mL^−1^, display similar trends under alkaline conditions, indicating comparable pH sensitivity.

**Fig. 3:**
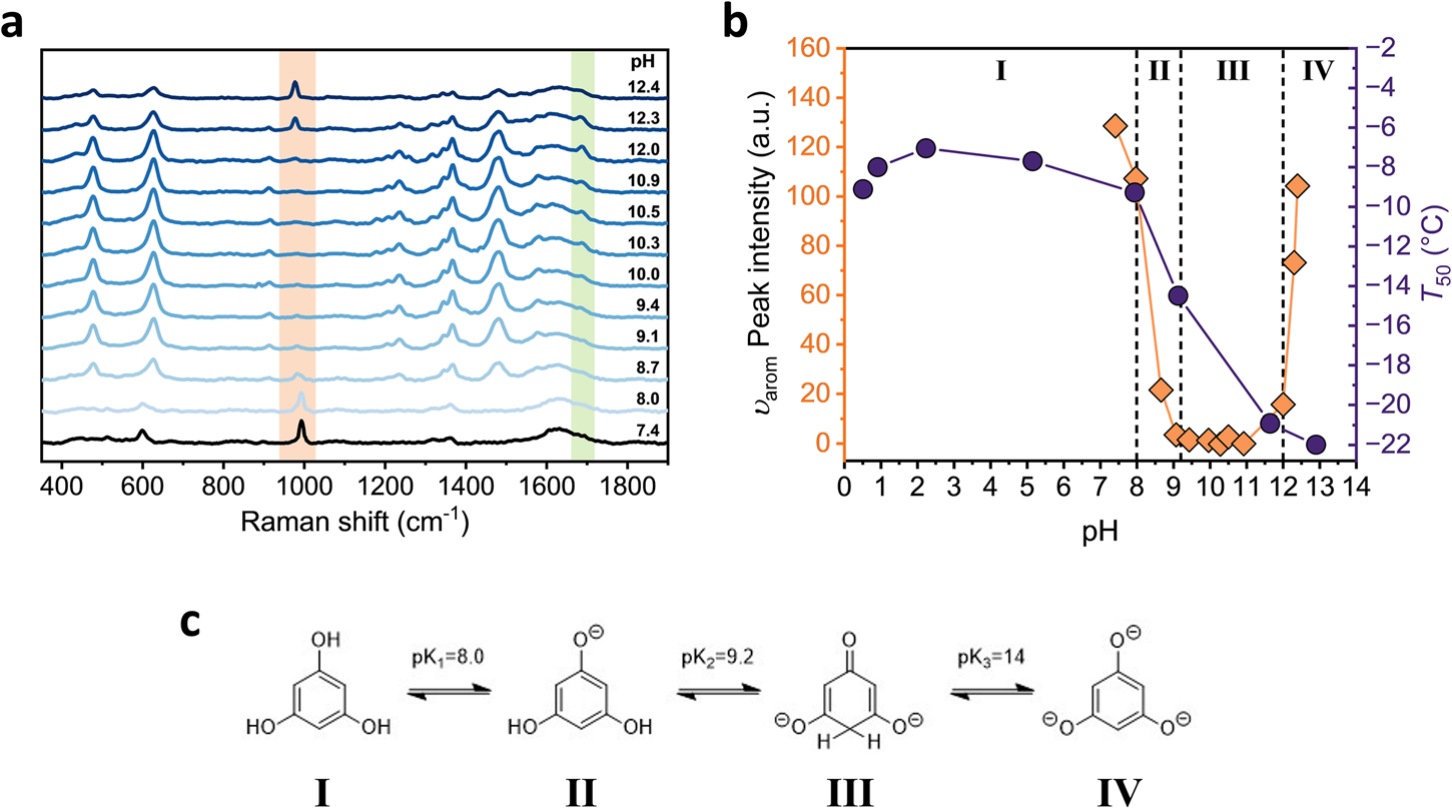
Raman and ice nucleation measurements of aqueous PGL solutions across different pH values. **a** Raman spectra of basic PGL solutions from pH 7 to pH 12.5. The orange- and green-shaded regions highlight the Raman signal of the in-plane ring vibration of the enol form and the carbonyl stretch vibration of the keto tautomer of PGL. **b** Intensity of the ~990 cm^−^^1^ aromatic Raman band as a function of pH, shown together with the corresponding *T*_50_ values from ice nucleation measurements. pK_a_ values of PGL reported by Wang et al.^47^ are indicated as dashed lines, and PGL tautomers are numbered in roman numerals. **c** Structures of the pH-dependent tautomeric equilibria of PGL.

To identify the mechanism underlying PGL’s ice nucleation activity and its change with pH, we probed PGL’s pH-dependent structure using Raman spectroscopy. PGL can undergo keto–enol tautomerism upon deprotonation of its hydroxyl groups^45^, and we hypothesize that these structural changes directly impair its ability to nucleate ice. Figure 3a shows Raman spectra of PGL solutions at pH values from 7 to 12.5. Neutral and mildly basic pH display vibrational signatures characteristic of the aromatic enol tautomer, including a strong signal at ~990 cm^−1^, corresponding to the in-plane ring vibration of the aromatic form^46^. This signal gradually weakens and disappears as the pH increases, replaced by bands associated with keto-like structures. An inverse trend appears in the C = O stretching region at ~ 1640 cm^−1^, supporting the shift in tautomeric shapes. Interestingly, at much higher pH values (≥12.3), the previous aromatic bands reemerge, consistent with a restoration of aromaticity. These spectroscopic changes are consistent with reported pK_a_ values of PGL, and correspond to sequential deprotonation and structural transitions between aromatic and non-aromatic tautomers^47^.

Correlating these spectroscopic insights with our ice nucleation data in Fig. 3b reveals that the deprotonation of hydroxyl groups and the loss of the aromatic signal coincide with a sharp decline in *T*_50_, directly linking ice nucleation activity to tautomeric structure. The tautomeric transition is illustrated in Fig. 3. At neutral pH, PGL predominantly exists in its aromatic enol form **I**, which enables high ice nucleation efficiency. Deprotonation at pK_1_ ≈ 8 yields a monoanionic, still aromatic species **II** with slightly reduced IN activity. A second deprotonation at pK_2_ ≈ 9.2 disrupts aromaticity, yielding a dianionic, non-aromatic keto-form **III**, consistent with the emergence of the ~1640 cm^−1^ signal and prior NMR studies reporting the formation of 3,5-dihydroxy-2,5-cyclohexadienone^47,48^. This structural transition coincides with the complete loss of measurable nucleation activity. Importantly, the loss of IN activity is not reversed by regaining aromaticity; instead, the fully deprotonated species at higher pH (species **IV**, pK_3_  >  12) highlights the importance of the hydroxyl groups and protonation state for efficient ice nucleation by PGL. However, readjusting the pH of fully deprotonated PGL to its initial pH enables the recovery of ice nucleation activity (Suppl. Fig. S3).

Together, these measurements reveal that PGL’s ice nucleating ability depends critically on its tautomeric balance and protonation state. Deprotonation of PGL’s hydroxyl groups induces structural rearrangements that eliminate aromaticity and disrupt the hydrogen bonding geometry required for ice formation. This establishes tautomerism and protonation equilibria as chemical switches that regulate access to nucleation-active molecular structures, especially for phenolic and polyhydroxylated compounds. Given that pH fluctuations can occur in clouds, biological fluids, and atmospheric aerosols, such equilibria can have a potentially strong influence on ice nucleation activity in real-world environments. The sensitivity of Raman spectroscopy to these structural changes highlights the value of vibrational signals in linking molecular structures to freezing behavior in systems where protonation equilibria are accessible.

### Chemical aging and phase transitions shape ice nucleator populations

Structural changes of PGL’s molecular composition significantly affect its ice nucleation activity. PGL solutions experience a decline in their nucleation activity within days, even when stored at preserving conditions. Visual inspection of aged solutions reveals the emergence of yellow discoloration, indicating structural changes associated with photosensitivity, oxidation and polymerization reactions typical for phenolic compounds^49,50^. Such reactions alter the tautomeric and hydrogen-bonding topology, deactivating PGL molecules and reducing their availability for ice-nucleating structures in both solution and crystalline material.

**Fig. 4:**
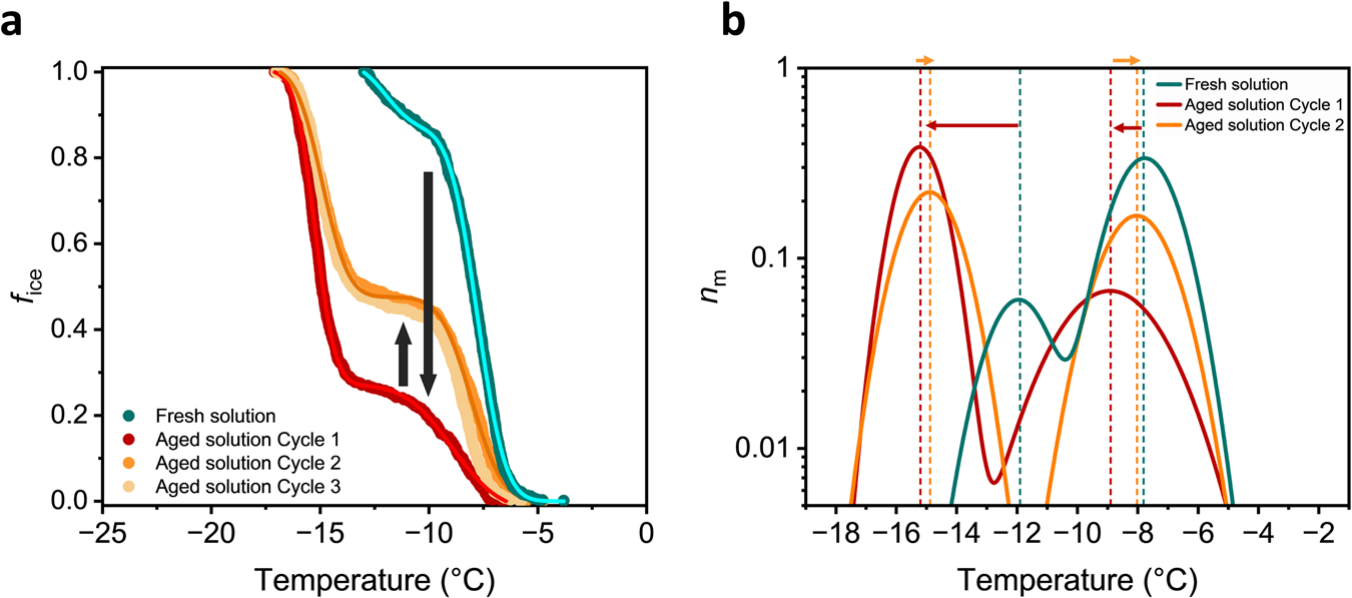
Effect of aging on the freezing activity of long-term stored PGL solutions. **a** *f*_ice_ of a 10 mg mL^−^^1^ PGL solution (green) when freshly prepared and after long-term storage (red). Consecutive freeze-thaw cycles partially restore activity (orange and yellow). Solid lines represent the optimal solution obtained by HUB analysis. **b** Normalized differential freezing spectra *n*_m_ obtained by HUB analysis of the *f*_ice_ plots in (**a**). *n*_m_ represents the distribution of heterogeneous ice nucleation temperatures of an IN sample.

Aging reduces both the number of active nucleators and the freezing modes as shown in Fig. 4a. Analysis of the IN subpopulations by the Heterogeneous Underlying-Based (HUB) method^51^, which utilizes a stochastic optimization algorithm to derive the differential freezing spectrum from experimental data, confirms that the bimodal distribution of INs is overall affected (Fig. 4b), with both modes shifted to lower temperatures. Structural aging reduces the initial freezing efficiency of the high temperature mode from −7.8 °C to −8.9 °C by ~56%, and diminishes the lower temperature mode at −12 °C to −15.2 °C (see Suppl. Table S1).

Interestingly, we observe a partial recovery of the ice nucleation activity of long-term aged PGL solutions upon freeze–thaw cycling. Subjecting aged solutions to a single freeze-thaw step restores approximately 19% of the higher freezing mode active around −8 °C, with no further changes after additional cycles. Reactivation of these highly efficient PGL INs was accompanied by precipitation of visible crystalline material in the previously clear solution (Suppl. Fig. S4). This suggests that freezing drives PGL past its solubility limit, promoting the formation of new microcrystalline domains that regain some nucleation efficiency lost through irreversible chemical modification by oxidation and polymerization. This response differs markedly from biological INs, such as those from bacteria, which typically lose activity upon repeated freeze–thaw cycling^43^. In contrast, PGL solutions show a partial restoration of nucleation efficiency due to the precipitation of newly formed, active PGL crystals, indicating that the observed activity is governed by reversible physicochemical processes rather than fragile supramolecular assemblies. The extent of this recovery depends directly on the remaining concentration of unreacted PGL in the aged solution. As aging proceeds, the amount of unreacted PGL is expected to decrease to levels that may eventually become too low to enable crystal formation. However, even after more than one year, crystallization in a 10 mg mL^−^^1^ solution is still observed, suggesting that complete loss of activity occurs on much longer timescales.

These observations show that PGL’s nucleation activity is dynamically reshaped by competing chemical and physical processes. Structural aging diminishes the IN populations, whereas cooling-induced phase transitions regenerate crystalline species, restoring activity. IN populations are therefore not static but evolves on experimental timescales, reflecting the interplay of chemical stability, solubility, and phase behavior.

### Solubility equilibrium determines the ice nucleation pathway of PGL

**Fig. 5:**
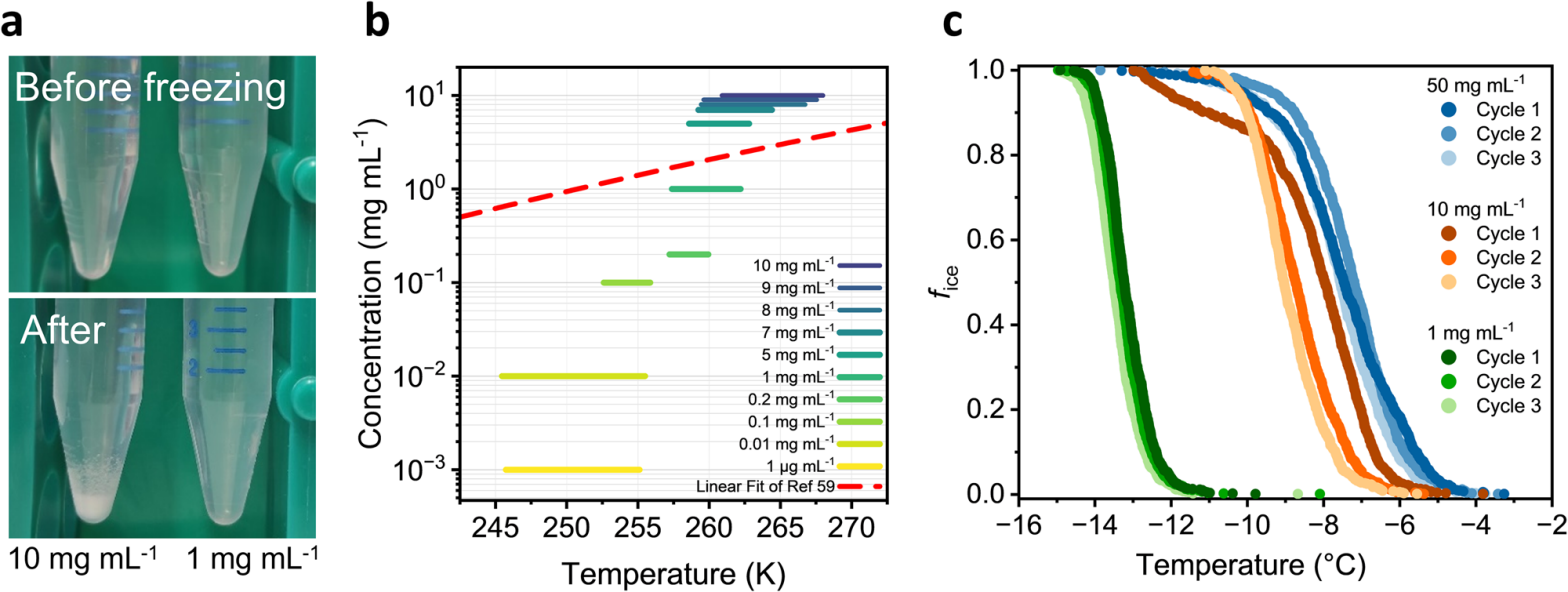
Freeze-thaw experiments of PGL solutions. **a** Pictures of PGL solutions before freezing and after thawing, showing the formation of precipitated crystals in 10 mg mL^−1^ solution, but not at 1 mg mL^−1^. **b** Freezing range of PGL solutions across different concentrations alongside the solubility threshold estimated by fitting solubility data of PGL reported by Zenkevich^59^ to the van’t Hoff equation (red line). **c** Fraction of ice (*f*_ice_) over three freeze-thaw cycles. No change is observed for undersaturated (1 mg mL^−1^) and supersaturated (50 mg mL^−1^) solutions, while distinct changes appear in solutions near the solubility limit (10 mg mL^−1^).

The concentration-dependent freezing behavior and temperature-driven phase transitions point to the existence of multiple ice nucleation pathways for PGL solutions. To identify their physical origin, we examined how PGL’s phase behavior relates to its ability to nucleate ice. For this, we examined PGL solutions after freeze–thawing, shown in Fig. 5a. The solubility of PGL in water is only around ~12 mg mL^−1^ ^52^, and solutions near this limit readily precipitate crystals upon cooling, as observed for aged solutions. Freeze-thawing a fresh 10 mg mL^−1^ solution confirms the formation of new PGL crystals upon cooling, whereas undersaturated solutions (1 mg mL^−1^) remain fully dissolved yet still display measurable activity. This suggest that the warmer temperature mode, which closely matches the freezing efficiency of crystalline PGL, originates from cooling-induced formation of crystals in solubility-critical solutions, while the ice nucleation activity of undersaturated solutions is based on less efficient dissolved structures. The absence of PGL crystals in undersaturated solution is further supported by the lack of sedimentation of crystals after prolonged equilibration or centrifugation and the absence of changes in *f*_ice_ that would be expected from crystal formation, as observed in solubility-critical solutions (Fig. 5c).

DLS measurements further confirm that solutions contain nanoscale PGL aggregates with hydrodynamic radii of *R*_h_ ~ 86 nm (Suppl. Fig. S5). Their size and scattering intensity decrease as pH increases, coinciding with the pH-dependent loss of nucleation activity, and eventually vanishing entirely under strongly basic conditions. These aggregates are hypothesized to consist of self-assembled molecular clusters that serve as nucleation sites, lowering the energetic barrier for ice formation. As the solution pH becomes alkaline, the tautomeric equilibrium shifts toward deprotonated PGL species, reducing the number of molecules capable of self-assembly due to electrostatic repulsion. Consequently, smaller aggregates are formed, as reflected by lower count rates and hydrodynamic radii, indicating that self-assembly is progressively disrupted by electrostatic repulsion. In addition, the concurrent loss of aromaticity, together with the inability to determine reliable *R*_h_ values at very low count rates, suggests that π-π interactions may contribute to PGL self-assembly. This suggests a nucleation pathway that is independent of bulk crystalline surfaces, in which nanoscale molecular assemblies in solution mediate ice nucleation.

The solubility threshold thus divides the system into two nucleation regimes. Modeling PGL’s solubility as a function of temperature using the van’t Hoff Eq. (1) (Fig. 5b) demonstrates that solutions with concentrations ≥5 mg mL^−1^ exceed the solubility threshold within their freezing range, consistent with crystal formation during cooling. In contrast, solutions at or below 1 mg mL^−1^ remain below the solubility limit, yet still nucleate ice effectively without the presence of detectable crystals, relying instead on nanoscale aggregates.

Upon multiple freeze-thaw cycles (Fig. 5c), both supersaturated and undersaturated PGL solutions maintain stable freezing profiles, indicating robust ice-nucleating structures in both dissolved and crystalline regimes. Notably, solubility-critical solutions (10 mg mL^−1^) experience a memory effect during freeze-thaw cycling: INs initially active at higher temperatures lose efficiency, while those active at lower temperatures become more active (Suppl. Fig. S6), as confirmed by statistical analysis of the freezing temperature evolution of each droplet over consecutive cycles (Suppl. Fig. S7). As the most efficient INs are lost, the average freezing temperature decreases and droplet freezing events become more uniformly distributed, further confirmed by HUB analysis (Suppl. Table S2). This suggest that the formation of new PGL crystals is a dynamic process and subject to fluctuations in crystalline IN site sizes and reorganization of crystal surfaces during formation. In addition, crystal sizes are influenced during the thawing step, as crystals dissolve back into solution when the samples are returned to room temperature.

Combined, our results support the existence of two distinct ice nucleation pathways: an efficient crystal-surface mechanism and a less efficient nanoscale solution aggregate mechanism. Which pathway operates is governed by the solubility equilibrium. At concentrations near or above the solubility threshold, ice nucleation is dominated by existing and newly precipitated PGL crystals, which provide surfaces for ice templating. This is in line with molecular simulations^42^, indicating templating on the crystal’s (010) face, which offers a favorable lattice match and hydrogen-bonding geometry for ice^42^. Precipitation of fresh PGL crystals is accompanied by broadening of the freezing spectrum, which reflects a distribution of crystal sizes and surface areas, consistent with our droplet freezing results for crystalline samples. At concentrations below the solubility limit, nucleation arises from dissolved PGL, forming aggregates that lower the nucleation barrier. Notably, HUB analysis of solutions below the solubility limit exhibit only one population, which shifts towards lower temperatures with decreasing concentration (Suppl. Fig. S8), consistent with the presence of concentration-dependent molecular PGL assemblies.

The pH-dependence, stemming from PGL’s keto-enol tautomerism, modulates both pathways, as observed in Fig. 2b. Deprotonation hinders aggregate formation by weakening intermolecular, likely π-mediated, interactions, while in the crystalline regime it suppresses crystal formation through tautomeric rearrangements^53^. The suppression of crystal formation is evident during cooling of solubility-critical solutions at strongly basic pH, where crystals no longer form. This behavior is consistent with deprotonation of PGL, which reduces the concentration of fully protonated aromatic PGL molecules required for crystallization. In addition, pre-existing crystals dissolve completely under strongly basic conditions.

These findings contrast with the usual classification of crystalline and soluble INs into separate categories and instead demonstrate that phase state and concentration can shift transitions between modes of activity in a reversible manner. This establishes an experimental framework for understanding concentration-dependent behavior in poorly soluble organic INs. Consequently, organic systems with similarly low solubility and a propensity for self-assembly are therefore expected to show comparable dual-pathway behavior.

### Classical nucleation modeling quantifies the size-activity relationships

The experimental data reveals two distinct classes of nucleators of different size dimensions: efficient microscale crystals active at warm temperatures and less efficient nanoscale aggregates active at lower temperatures. To determine whether these species are consistent with physically reasonable nucleating areas, we used the Heterogeneous Ice Nucleation Temperature (HINT) implementation of classical nucleation theory to relate nucleation temperature to the size of PGL’s ice binding domains^54^. The calculated nucleation temperatures increase steeply with lateral dimensions up to approximately 18 × 18 nm^2^, with little further change for larger surfaces (Suppl. Fig. S9). Below this size threshold, already small changes in size have tremendous effects on the nucleation temperature. While tested crystals show microscale sizes, coherent ice-binding sites may be smaller, whose dimensions and constitutions are sculptured by surface irregularities and crystallographic defects. In the dissolved regime, HINT analysis indicates that nanoscale domains with surface areas of approximately 4 × 4 to 7 ×7 nm^2^ are sufficient to account for the observed activity, consistent with concentration-dependent assembly of aggregates.

These calculations show that the two experimentally identified nucleating species are not only qualitatively distinct but also quantitatively compatible with classical nucleation theory. Crystal surfaces provide larger effective areas needed for high freezing efficiency, and small aggregates provide the restricted area expected for activity in the colder temperature range. The mechanistic picture that emerges – dual nucleation pathways governed by solubility, tautomerism, and phase behavior – is therefore both chemically and thermodynamically coherent.

## Conclusion

Our study reveals that PGL does not behave as a single, well-defined ice nucleator but as a dynamically evolving system, whose activity is governed by an intricate interplay of chemical and physical equilibria. Specifically, tautomeric and ionization states, solubility, and phase behavior critically shape nucleation efficiency. Ice nucleation emerges from two distinct yet reversible pathways linked to PGL’s phase behavior: Crystal surfaces dominate when the solubility limit is exceeded, creating large, flat domains capable of nucleating ice at high subzero temperatures, whereas nanoscale aggregates persist in undersaturated solutions and act as less efficient nucleators. Aging and high pH both disrupt the aromatic ice-nucleating geometry and suppress activity by altering molecular aggregation and preventing crystallization. Freeze-thaw cycling restores activity by driving recrystallization. Classical nucleation theory quantitatively resolves these two families of nucleators, linking the observed freezing temperatures to physically plausible domain sizes.

These findings challenge the conventional classification of ice nucleators into crystalline versus soluble categories, demonstrating instead that phase state and concentration dynamically modulate modes of activity. This reveals that even structurally simple organic compounds can exhibit complex freezing behavior through reversible transitions between dissolved and crystalline phases. A complete understanding therefore requires consideration of both chemical and physical equilibria, particularly in environmentally and biologically relevant contexts where temperature, pH, and concentration can vary significantly over relevant timescales, and cannot rely on simplified models treating ice nucleation as dependent solely on surface properties or molecular structure. Further, this duality challenges the prior water-only monolayer hypothesis^40^, suggesting instead that mixed-phase contributions underlie PGL’s exceptional ice nucleation in crystalline state.

The structural features that determine phloroglucinol’s activity are shared by many naturally occurring phenolic and polyhydroxylated compounds. Consequently, the mechanistic principles described here likely apply broadly to this extensive and environmentally relevant class of organic ice nucleators, which may exhibit similar equilibria between aromatized, deprotonated, aggregated, and crystalline forms. Our combined experimental and modeling approaches therefore provide a generalizable framework for linking molecular-scale structure, chemistry, and phase transitions to freezing behavior in complex systems.

## Methods

### Materials

Pure water was obtained from Millipore Milli-Q® Integral 3 water purification system (Merck Chemicals GmbH, Darmstadt, Germany), autoclaved at 121 °C for 15 min, and filtered through a 0.1 µm bottle top filtration unit (VWR International GmbH, Darmstadt, Germany). Phloroglucinol dihydrate was purchased from Sigma-Aldrich (99%, Darmstadt, Germany) and Thermo Scientific (99%, Waltham, MA, USA). The crystallinity of the samples was verified by powder X-ray diffraction (XRD). The recorded diffraction pattern (Suppl. Fig. S10) agrees with reported XRD data for Phloroglucinol dihydrate^55^, confirming the crystalline structure of the material. NaOH and HCl were purchased from Sigma-Aldrich (Darmstadt, Germany).

### TINA Measurements

Ice nucleation experiments were performed using the high-throughput Twin-plate Ice Nucleation Assay (TINA), which allows to study ice nucleation under immersion conditions and has been described in detail elsewhere^56^. In a typical experiment, the investigated IN sample was serially diluted by a liquid handling station (epMotion ep5073, Eppendorf, Hamburg, Germany). 96 droplets (droplet volume: 3 µL) per dilution were placed on two 384-well plates and tested with a continuous cooling-rate of 1 °C min^−1^ from 0 °C to −30 °C. The droplet freezing events were detected by two infrared cameras (Seek Thermal Compact XR, Seek Thermal Inc., Santa Barbara, CA, USA). The uncertainty in the temperature of the setup was ± 0.2 °C. The temperature at which 50% of droplets in the assay have frozen, *T*_50_, were used as a quantitative measure of ice nucleation efficiency. The cumulative number of INs per unit mass *N*_m_(*T*) were calculated from the obtained fraction of frozen droplets *f*_ice_ using the Vali formula^44^. Experiments were performed at least three times on independent samples. Background freezing of pure water in our system occurred at −22.5 °C. A droplet volume of 3 µL was chosen for the freezing experiments, as it represents the minimal volume reliably detectable by the employed IR cameras. This volume also provides an appropriate temperature window for studying heterogeneous ice nucleation, as droplets of this size typically undergo homogeneous freezing near −40 °C^57,58^. Importantly, the heterogeneous nucleation temperatures of PGL were independent of droplet volume over the range of 3–30 µL (Suppl. Fig. S11).

For measurements of PGL dihydrate crystals (solubility in water: ~12 mg mL^−1^)^52^, an oversaturated solution of 50 mg mL^−1^ was created. Dilution series of PGL were created from a highly saturated 10 mg mL^−1^ solution. For freeze-thaw experiments, aqueous Phloroglucinol samples were prepared freshly, and 768 droplets of 3 µL volume were placed on two 384-well plates. After being cooled down to −30 °C, the samples were allowed to thaw at room temperature before the next measurement. At least 3 consecutive freeze-thaw cycles were performed for each experiment.

### Crystal characterization

Powder XRD was performed using a Rigaku SmartLab X-ray diffractometer in Bragg-Brentano geometry with Cu Kα radiation (λ = 0.154059 nm) generated from a rotating anode operated at 45 kV and 200 mA. A 10 mm limitation slit was used. Diffraction data were collected at ambient temperature using a HyPix-3000 2D detector over a 2θ range of 10° to 40° with a step size of 0.01° and a scan speed of 1° min^−1^.

The particle size distribution for PGL dihydrate crystals was determined by optical microscopy using the optical imaging mode of the Oxford Instruments alpha300Ri Raman microscope. Crystals were dispersed on glass microscope slides, and images were acquired over an area of 2 × 3 cm using a 20x objective. To optimize image contrast for particle analysis, the microscope’s internal illumination was disabled, and imaging was performed under ambient room lighting. Crystal diameters and areas were quantified by automated image analysis using WiTec Particle Scout software.

Further crystallographic characterization via high-resolution imaging was technically not feasible. Attempts to analyze the samples using SEM and TEM under high vacuum led to rapid sublimation of the PGL dihydrate crystals, preventing reliable assessment of surface topography and face-specific morphology.

### Raman spectroscopy

Twelve 10 mg mL^−1^ solutions of phloroglucinol were prepared from solid phloroglucinol dihydrate. 6 M NaOH was prepared and added to each solution to increase the pH. pH was measured by an H^+^-ion-selective electrode. Solutions were prepared at each of the following pH values: 7.41, 7.97, 8.66, 9.07, 9.43, 9.96, 10.28, 10.50, 10.92, 12.00, 12.30, 12.39. Raman spectra were taken using the Oxford Instruments alpha300Ri confocal research microscope. For each pH solution, 20 μL was pipetted onto a glass slide and situated above the objective of the microscope. Measurements were taken using a 532 nm laser with 36.5 mW of power. Each spectrum reported is an average of 30 accumulations, each with a 2-second integration time.

### Dynamic light scattering

The hydrodynamic radii, *R*_h_, of 5 mg mL^−1^ phloroglucinol solutions, adjusted to the desired pH, were determined using dynamic light scattering (DLS). Measurements were performed on an ALV spectrometer equipped with a He-Ne laser (wavelength of 632.8 nm) as the light source. Prior to measurements, solution pH was adjusted using 1 N NaOH, and samples were filtered through 0.45 µm PES membrane syringe filters to remove dust. DLS measurements were performed at 20 °C with a scattering angle of 90°.

### Heterogeneous Underlying Based (HUB) Analysis of IN subpopulations

The Heterogeneous Underlying-Based (HUB) method^51^ was utilized for the identification and quantification of IN subpopulations that constitute the experimental cumulative freezing spectra and the fraction of ice. This method uses a stochastic optimization technique to extract the underlying distribution of heterogeneous ice nucleation temperatures *P*_u_(*T*) that describes the characteristic freezing temperatures of all INs in a sample. For this, the HUB-backward code available as a Python code (https://github.com/Molinero-Group/underlying-distribution) was used to compute the differential freezing spectra *n*_m_(*T*), representing *P*_u_(*T*), from the cumulative freezing spectra *N*_m_(*T*) or the fraction of ice *f*_ice_ obtained from TINA experiments. *P*_u_(*T*) is assumed to be a linear combination of normalized Gaussian distribution functions *P*_i_(*T*) that represents a distinct number of subpopulations *p* of the weights *c*_i_ that give ${\sum }_{i=1}^{p}{c}_{i}=1$. Each subpopulation *P*_i_(*T*) is further characterized by its characteristic freezing temperature mode *T*_mode,i_ and the spread of the temperature distribution *s*_i_. The experimentally obtained data is interpolated through a spline and smoothed with a Savitzky-Golay filter of first polynomial order with a default value of 3 for the length of the filter window. The mean squared error MSE defines the accuracy of the determined set of parameters for the distribution function. For further analysis, optimized results with the lowest MSE were selected.

### Calculation of solubility limits

The solubility data of phloroglucinol reported by Zenkevich et al.^59^ were fitted to the van’t Hoff equation

1 $$S\left(T\right)={S}_{0}\,\exp \left(-\frac{\Delta {H}_{{{\rm{sol}}}}}{{RT}}\right)$$

where *S*(*T*) is the solubility (in wt%) at temperature *T*, *S*_0_ is the pre-exponential factor corresponding to the hypothetical solubility at infinite temperature, Δ*H*_sol_ is the enthalpy of dissolution, *R* is the universal gas constant, and *T* is the absolute temperature in Kelvin (Suppl. Fig. S12). The fit yielded *S*_0_ = 7 × 10^7 ^wt% and Δ*H*_sol_ = 42,460 J mol^−1^ ( ≈ 10.15 kcal mol^−1^). The solubility *S* was converted to the concentration *C* in mg mL^−1^ using

2 $$C=S\times 10\times \rho$$

where *ρ* is the density of the solution (assumed to be 1 g mL^−1^ for water). Under this assumption, 1 wt% corresponds to 10 mg mL^−1^.

### Prediction of Heterogeneous Ice Nucleation Temperatures

The Heterogeneous Ice Nucleation Temperature (HINT) algorithm^54,60^ is a numerical implementation of classical nucleation theory^61,62^ (CNT) developed to predict the temperature at which ice nucleates on a given surface. HINT has been applied to predict the size of active ice-nucleating proteins (INPs) in bacteria and fungi^43,63,64^, as well as the effective nucleating area of wedge-shaped topographic defects^65^. In this framework, the free-energy barrier for forming a critical ice nucleus, Δ*G*, is evaluated by incorporating the geometry of the nucleating surface and the interfacial binding free energy with ice Δ*γ*_bind_. HINT integrates the geometric parameters, such as the nucleus curvature, contact angle, and wedge angle, into the CNT expression for Δ*G*, and identifies the minimum free-energy pathway for ice formation. The algorithm relies on experimentally determined thermodynamic and kinetic properties of water, including the temperature-dependent self-diffusion coefficient *D*(*T*), the chemical potential difference between liquid and ice Δ*μ*(*T*), and the ice–liquid surface tension *γ*_ice–liquid_(*T*). Using these quantities, HINT computes both the critical free-energy barrier Δ*G* and the kinetic prefactor *A*(*T*), and determines the heterogeneous freezing temperature, *T*_het_, as the point where the calculated nucleation rate equals the experimental rate *J*_exp_ = 10^5^ cm^−^^3^ s^−1^, corresponding to a cooling rate of approximately 1 °C min^−1^ ^66–68^.

## Supplementary information

Supplementary Information

Description of Additional Supplementary Files

Supplementary Data 1

## Data Availability

All data generated or analyzed during this study are included in this published article and its supplementary information files. Source data for all figures are provided in Suppl. Data 1.
