# Supplementary material for: Chemical and physical equilibria shape dual ice-nucleation pathways in an organic crystal
Source: Commun Chem. 2026 Jun 6;9:195. doi: 10.1038/s42004-026-02086-4 (PMC13242512; doi:10.1038/s42004-026-02086-4)
Supplement: Supplementary file 1 — Supplementary Information [file 42004_2026_2086_MOESM1_ESM.pdf]

Supplementary Information

Chemical and Physical Equilibria Shape Dual Ice-Nucleation Pathways in an Organic Crystal

Authors: Galit Renzer[1], Dawson Bell[2], Ingrid de Almeida Ribeiro[3], Kaden Shaw[2], Mischa Bonn[1], Valeria Molinero[3], Konrad Meister[1,2*]

Affiliations:

[1]Max Planck Institute for Polymer Research, 55128 Mainz, Germany

[2]Department of Chemistry and Biochemistry, Boise State University, 83725 Boise, ID, USA

[3]Department of Chemistry, The University of Utah, 84112 Salt Lake City, UT, USA

*To whom correspondence may be addressed. Email: meisterk@mpip-mainz.mpg.de

Table of Content:

Supplementary Figures and Tables....................3

References....................16

Supplementary Figures and Tables

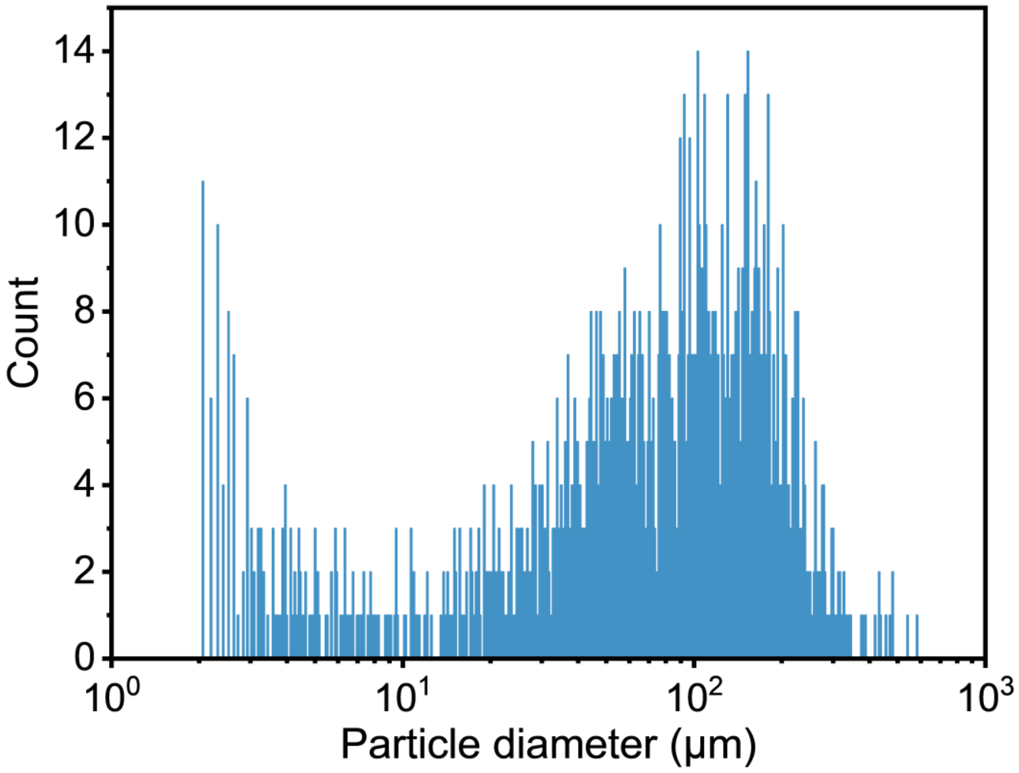

Supplementary Figure S1: Particle size distribution of PGL crystals. The histogram shows the particle diameters in counts. The distribution exhibits a mean particle size of 98 ± 79 μm, indicating a broad spread in particle sizes.

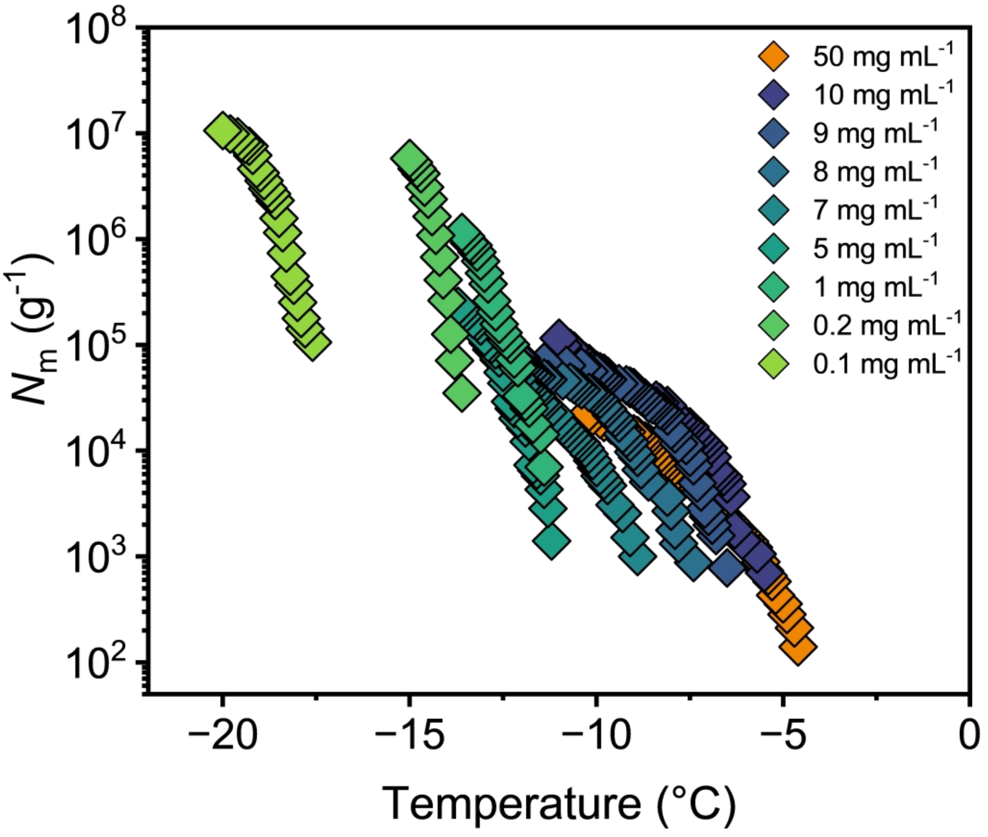

Supplementary Figure S2: Cumulative number of ice nucleation sites per unit mass (N_m) for the dilution series of PGL, ranging from 50 to 0.1 mg mL^{-1}.

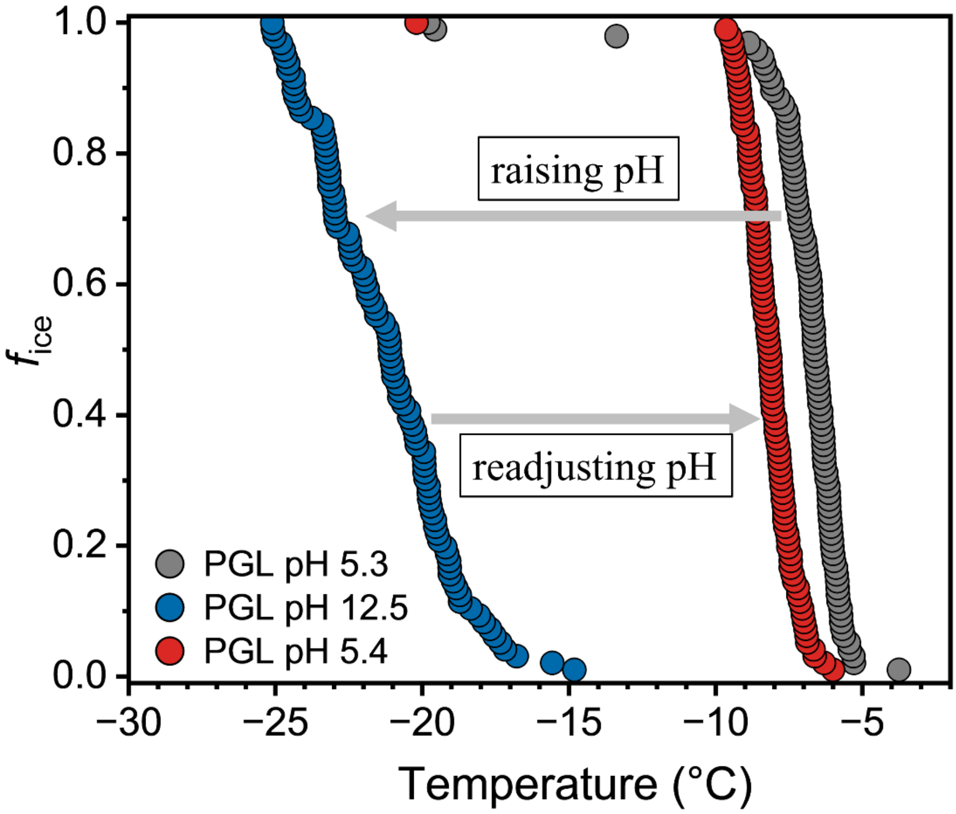

Supplementary Figure S3: Freezing experiments of a 10 mg mL^{-1} PGL solution showing the fraction of ice, f_{ice}, as a function of solution pH. The decline in activity caused by increasing the solution pH with NaOH is largely reversed upon readjustment to its original pH using HCl. The slight shift toward lower freezing temperatures compared to the initial activity results from sample dilution and additional colligative freezing point depression due to the added NaOH and HCl during pH adjustment.

Supplementary Table S1: Parameter of the normalized distribution functions of the fraction of ice f_{ice} of fresh and aged Phloroglucinol solutions obtained through the HUB method[1] using two Gaussian subpopulations. T_{mode} is the mode of the Gaussian distribution of heterogeneous ice nucleation temperatures, s is the spread of T_{mode}, and c the fraction of the subpopulation. The mean squared error (MSE) describes the quality of the found optimized solution for the experimental fraction of ice.

Label	$T_{mode,1}$ (°C)	s_1	c_1	$T_{mode,2}$ (°C)	s_2	c_2	MSE
**Fresh PGL solution**	-7.77	1.01	0.85	-11.95	1.00	0.15	0.00004
**Aged PGL solution Cycle 1**	-8.92	1.69	0.29	-15.23	0.74	0.71	0.0001
**Aged PGL solution Cycle 2**	-8.04	1.12	0.47	-14.89	0.95	0.53	0.0001
**Aged PGL solution Cycle 3**	-8.43	0.93	0.45	-14.90	0.86	0.55	0.0001

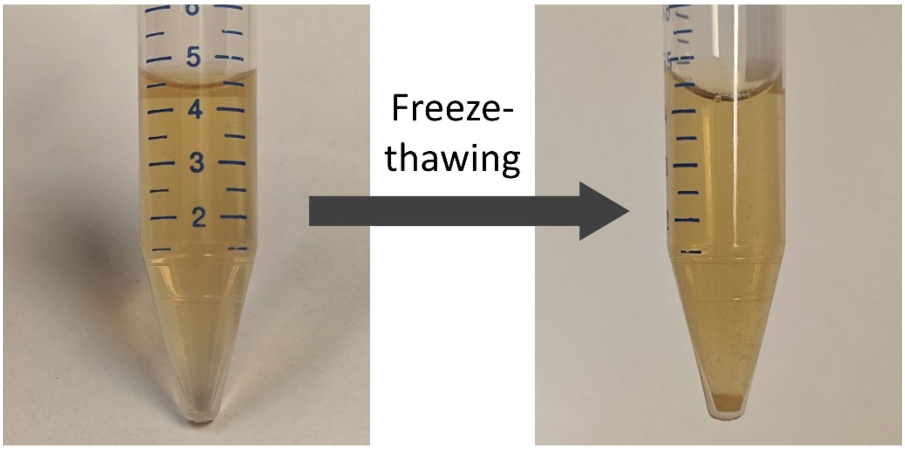

Supplementary Figure S4: Aged PGL solution with crystal formation after freeze-thawing.

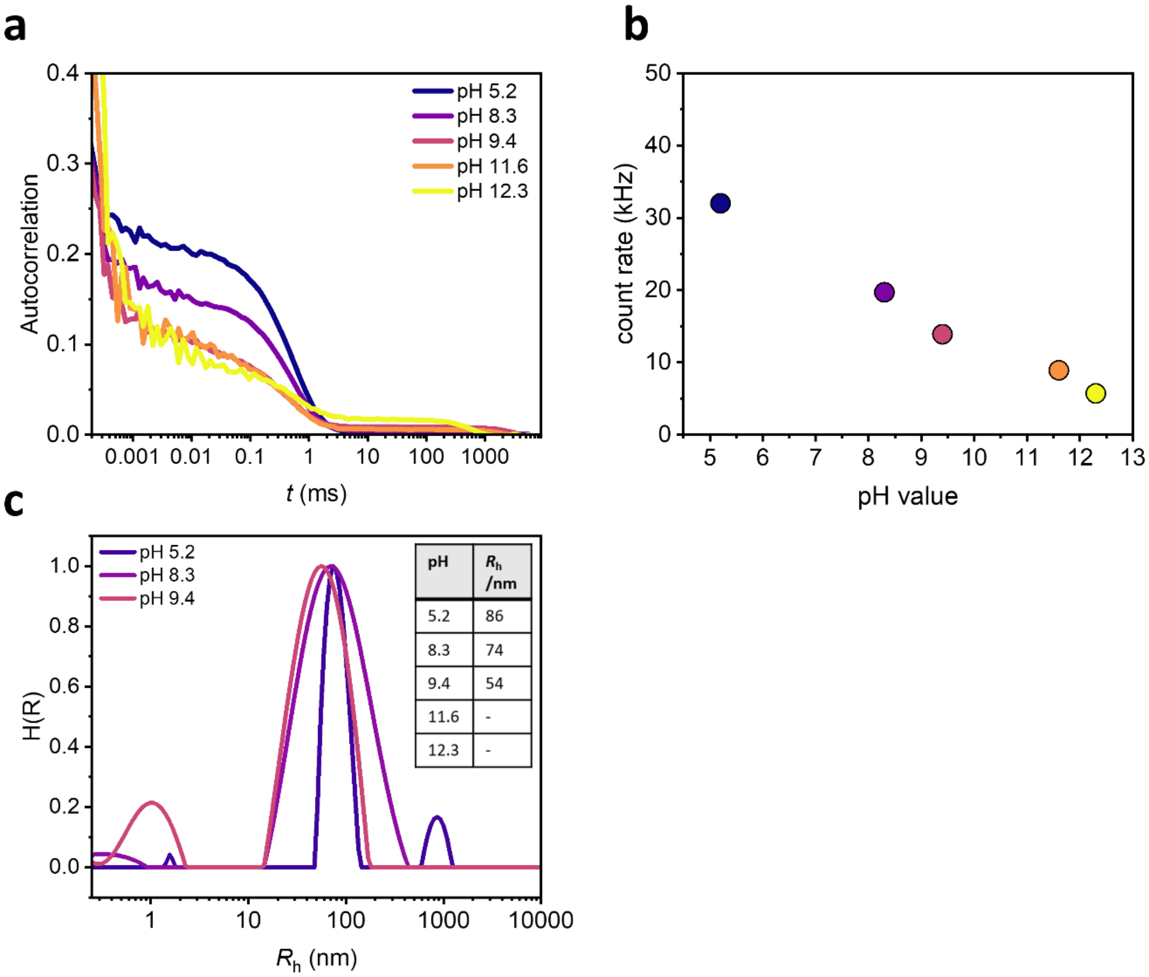

Supplementary Figure S5: Dynamic light scattering of Phloroglucinol (5 mg mL^{-1}) under basic pH conditions. **a** Autocorrelation functions and **b** corresponding count rates of scattered light, both decreasing with increasing pH. **c** Intensity-weighted distribution of hydrodynamic radii R_h, showing the average R_h of phloroglucinol assemblies at different pH levels. For pH 11.6 and 12.3, the scattering intensity was too low to obtain reliable size distributions.

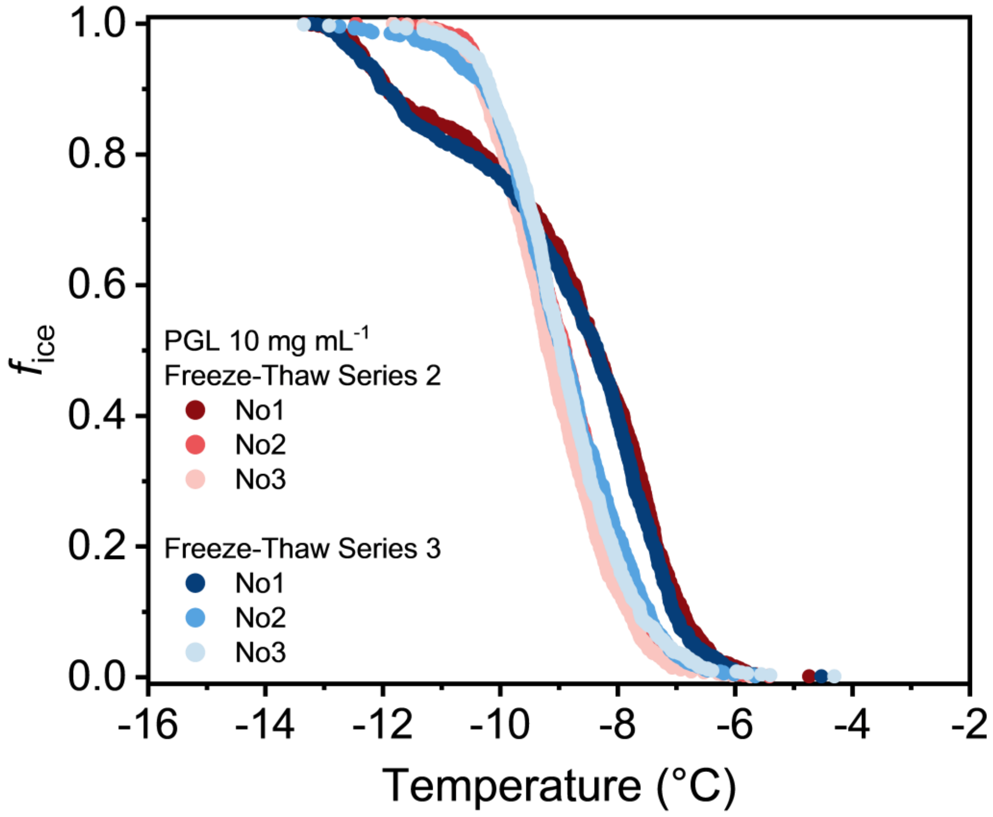

Supplementary Figure S6: Freeze-Thaw Experiments of 10 mg mL^{-1} PGL solutions.

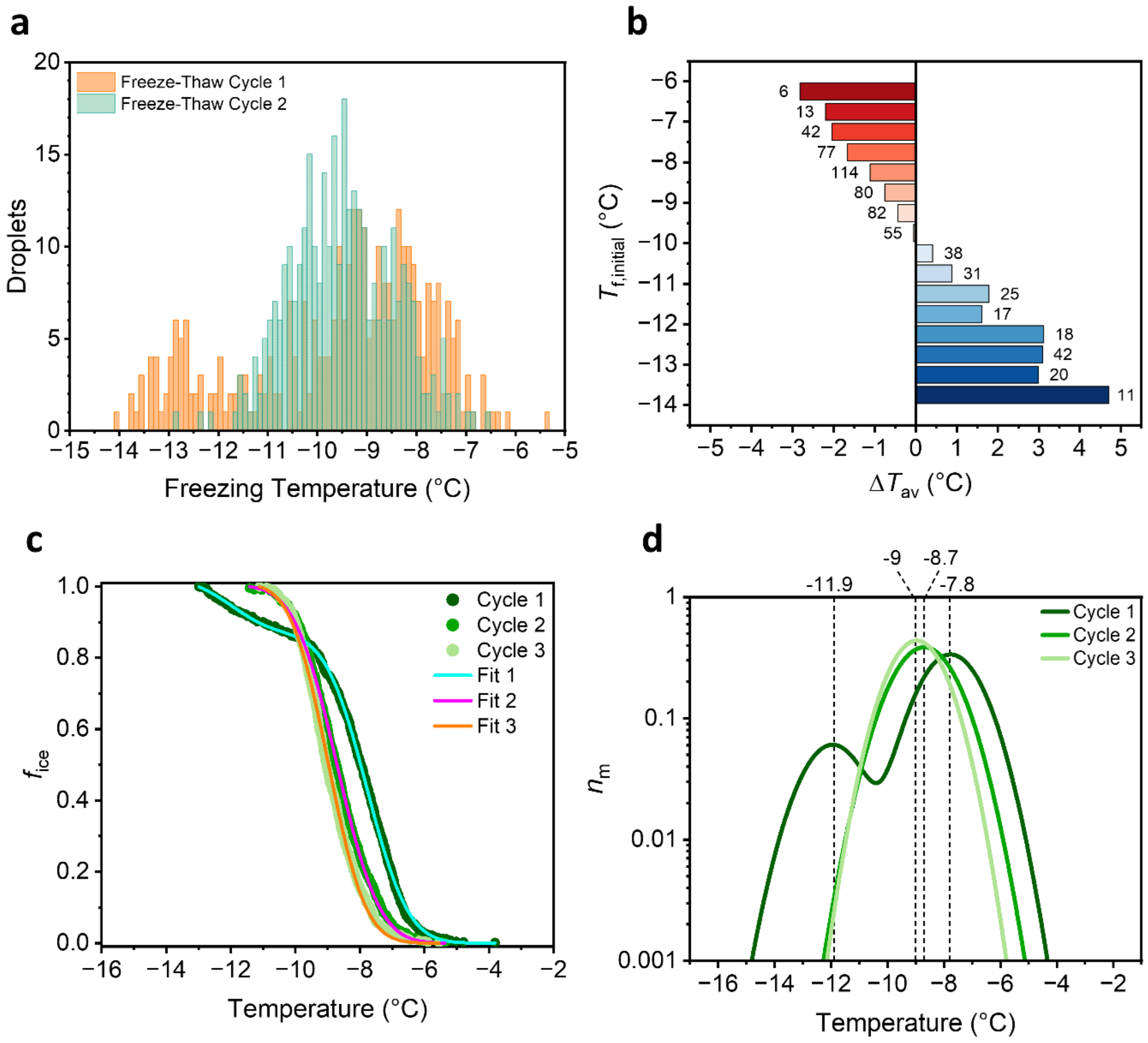

Supplementary Figure S7: Droplet freezing statistics of freeze-thaw cycled 10 mg mL^{-1} PGL solutions. **a** The initial freezing temperature $T_{f,initial}$ for each droplet (yellow) is shifted after second freeze-thawing (green). **b** Average shift in freezing temperature ΔT_{av} of all droplets initially active in the same 0.5°C increment after second freeze-thawing. The number of droplets in each increment is labeled beside the bars. **c** Optimized solutions of all f_{ice} plots obtained in the freeze-thaw experiment by assuming that the differential spectrum is a linear combination of Gaussian subpopulations. **d** Normalized distribution functions that represent the corresponding differential spectra n_m(T).

Supplementary Table S2: Parameter of the normalized distribution functions of the fraction of ice f_{ice} of Phloroglucinol solutions subjected to freeze-thaw cycles represented with 2 or 1 subpopulations. T_{mode} is the mode of the Gaussian distribution of heterogeneous ice nucleation temperatures, s is the spread of T_{mode}, and c the fraction of the subpopulation. The mean squared error (MSE) describes the quality of the found optimized solution for the experimental fraction of ice.

Label	$T_{mode,1}$ (°C)	s_1	c_1	$T_{mode,2}$ (°C)	s_2	c_2	MSE
**Cycle 1**	-7.77	1.01	0.85	-11.95	1.00	0.15	0.00004
**Cycle 2**	-8.7	1.03	1.00	-	-	-	0.0001
**Cycle 3**	-8.97	0.91	1.00	-	-	-	0.0001

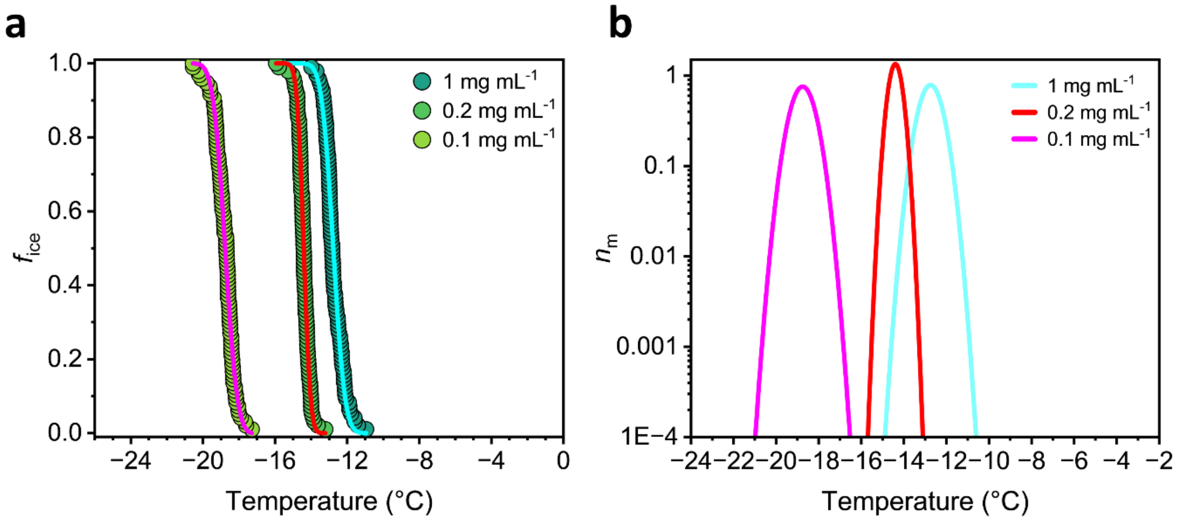

Supplementary Figure S8: HUB analysis of PGL solutions below the solubility limit. **a** The fraction of ice (f_{ice}) of 1, 0.2, and 0.1 mg mL^{-1} PGL solutions. Solid lines represent the optimal solution obtained by HUB analysis. **b** Normalized differential freezing spectra n_{m} representing the distribution of heterogeneous ice nucleation temperatures of 1mg mL^{-1} (-12.8 ± 0.5°C), 0.2 mg mL^{-1} (-14.4 ± 0.3°C), and 0.1 mg mL^{-1} (-18.8 ± 0.5°C).

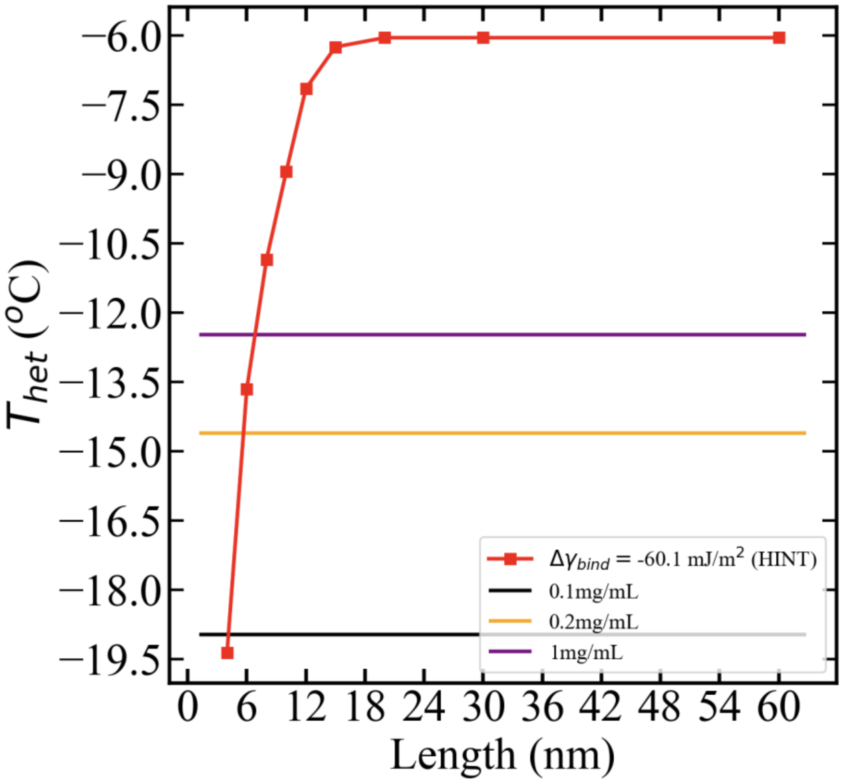

Supplementary Figure S9: Predicted ice nucleation temperatures as a function of the ice-binding site size. Ice nucleation temperatures were estimated using classical nucleation theory as implemented in the HINT algorithm, which calculates the critical free-energy barrier for ice formation on a given surface. The analysis considers square surfaces formed by the (010) crystal face, characterized by an interfacial binding free energy difference $\Delta\gamma_{\mathrm{bind}}$ between the phloroglucinol (PGL) surface and ice, normalized per unit area. The value of $\Delta\gamma_{\mathrm{bind}}$ was adjusted such that the surface is not limited by finite size effects and can nucleate ice at the highest experimental temperature observed for PGL dihydrate (−6 °C). The T_{50} values obtained from the experiments are shown for solutions of 1 mg mL^{-1} (black), 0.2 mg mL^{-1} (yellow), and 0.1 mg mL^{-1} (purple).

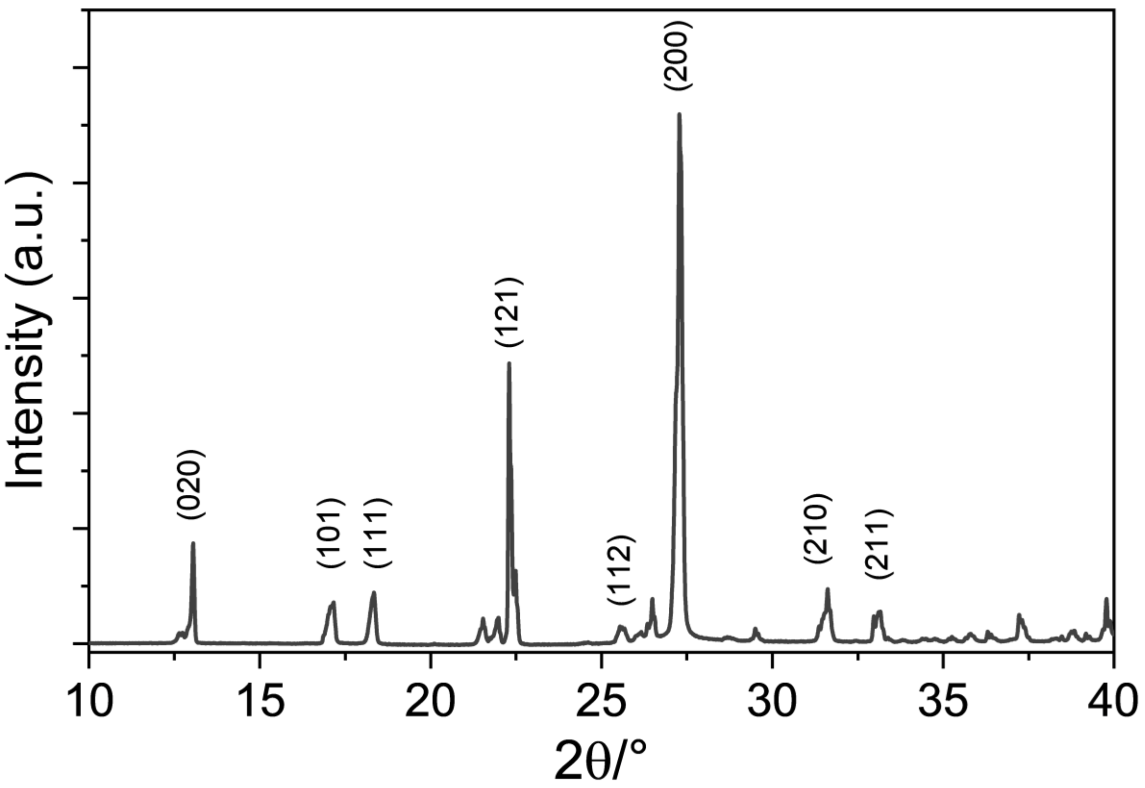

Supplementary Figure S10: Powder X-ray diffraction pattern of Phloroglucinol dihydrate crystallizing in the orthorhombic space group *Pnma* (a = 6.621 Å, b = 13.561 Å, c = 8.046 Å)[2].

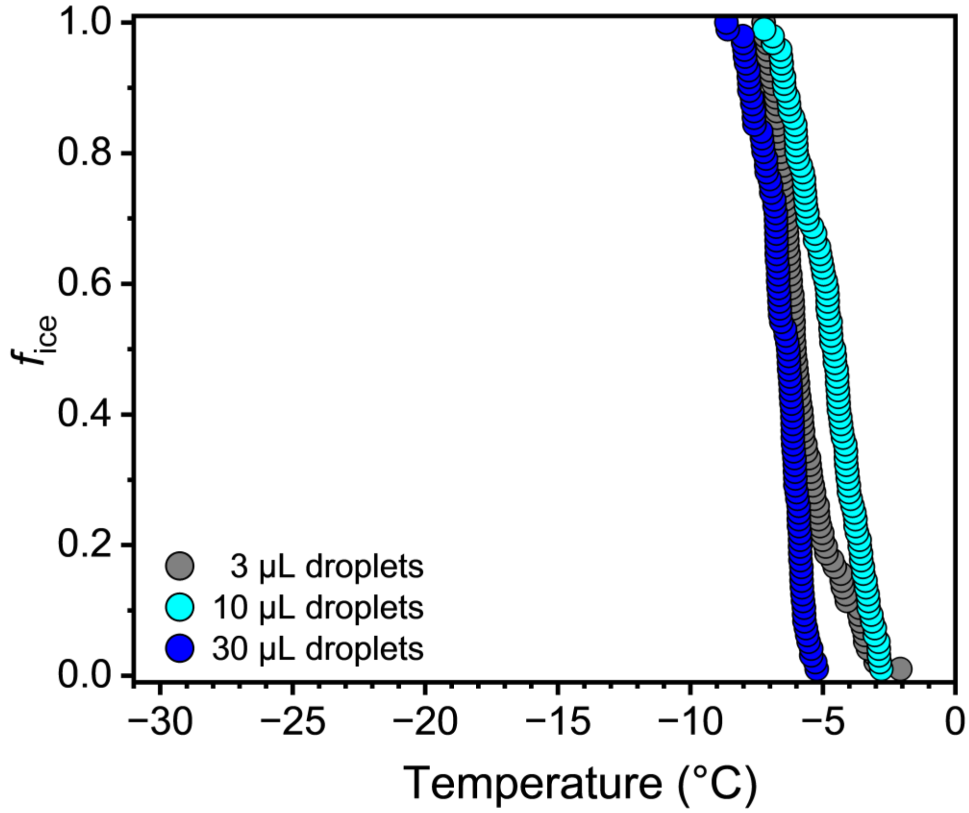

Supplementary Figure S11: Fraction of frozen droplets as a function of temperature for a PGL solution (10 mg mL^{-1}), measured at different droplet volumes (3, 10, and 30 µL).

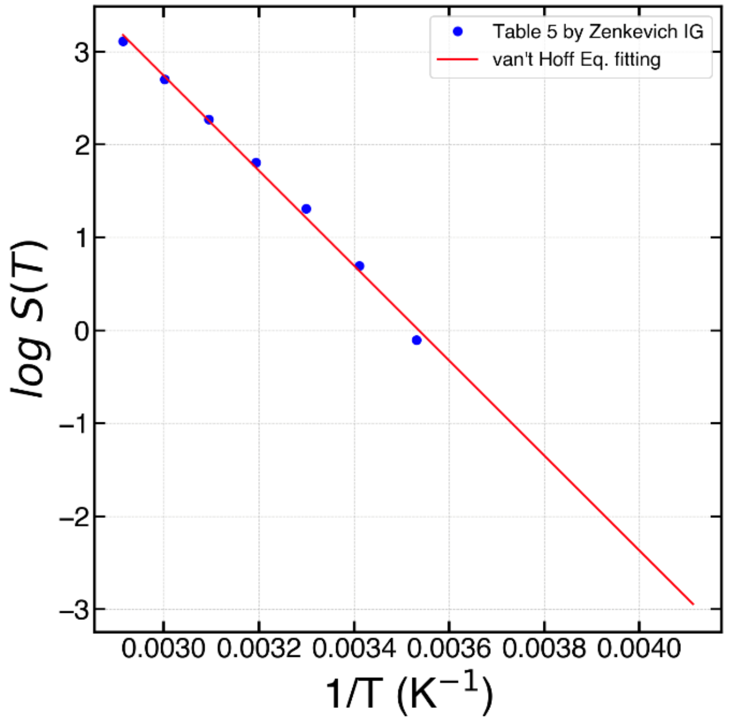

Supplementary Figure S12: Solubility data of phloroglucinol reported by Zenkevich[3] fitted to the van't Hoff equation.

References

1. de Almeida Ribeiro, I., Meister, K. & Molinero, V. Hub: A method to model and extract the distribution of ice nucleation temperatures from drop-freezing experiments. *Atmos. Chem. Phys.* **23,** 5623-5639 (2023).
2. Braun, D. E., Tocher, D. A., Price, S. L. & Griesser, U. J. The complexity of hydration of phloroglucinol: A comprehensive structural and thermodynamic characterization. *J. Phys. Chem. B* **116,** 3961-3972 (2012).
3. Zenkevich, I. G. Use of recurrence relations for approximating properties of any homologs of organic compounds. *Russ. J. Gen. Chem.* **76,** 1742-1752 (2006).
