# Supplementary material for: Chemical and physical equilibria shape dual ice-nucleation pathways in an organic crystal
Source: Commun Chem. 2026 Jun 6;9:195. doi: 10.1038/s42004-026-02086-4 (PMC13242512; doi:10.1038/s42004-026-02086-4)
Supplement: Supplementary file 2 — Description of Additional Supplementary Files [file 42004_2026_2086_MOESM2_ESM.pdf]

# Description of Additional Supplementary Files:

**File:** Supplementary Data 1

**Description:** Experimental data for measurements presented in the main text and the supplementary information

Datasets of each figure (including all panels) are provided in separate tabs in the Excel file.
